# Non-invasive pressure difference estimation from PC-MRI using the work-energy equation

**DOI:** 10.1016/j.media.2015.08.012

**Published:** 2015-12

**Authors:** Fabrizio Donati, C. Alberto Figueroa, Nicolas P. Smith, Pablo Lamata, David A. Nordsletten

**Affiliations:** a King’s College London, Department of Biomedical Engineering and Imaging Sciences, St. Thomas’ Hospital, 4th floor Lambeth Wing, The Rayne Institute, London SE1 7EH, United Kingdom; b University of Michigan, North Campus Research Complex, 2800 Plymouth Road, Ann Arbor, MI 48105, United States; c University of Auckland, Engineering School Block 1, Level 5, 20 Symonds St, Auckland 101, New Zealand; d University of Oxford, Department of Computer Science, Wolfson Building, Parks Road, Oxford OX1 3QD, United Kingdom

**Keywords:** 4D flow, PC-MRI, Pressure differences estimation, Work-energy equation, Aortic flow

## Abstract

Pressure difference is an accepted clinical biomarker for cardiovascular disease conditions such as aortic coarctation. Currently, measurements of pressure differences in the clinic rely on invasive techniques (catheterization), prompting development of non-invasive estimates based on blood flow. In this work, we propose a non-invasive estimation procedure deriving pressure difference from the work-energy equation for a Newtonian fluid. Spatial and temporal convergence is demonstrated on *in silico* Phase Contrast Magnetic Resonance Image (PC-MRI) phantoms with steady and transient flow fields. The method is also tested on an image dataset generated in silico from a 3D patient-specific Computational Fluid Dynamics (CFD) simulation and finally evaluated on a cohort of 9 subjects. The performance is compared to existing approaches based on steady and unsteady Bernoulli formulations as well as the pressure Poisson equation. The new technique shows good accuracy, robustness to noise, and robustness to the image segmentation process, illustrating the potential of this approach for non-invasive pressure difference estimation.

## Highlights

• A novel semi-automatic method for the estimation of pressure differences in cardiovascular compartments from dense velocity fields (4D flow MRI data).
• The approach relies on the work-energy principle, and removes the need for second order spatial derivatives.
• Pressure differences are evaluated directly from the 4D flow data, without the need of any computational mesh.
• The method shows good accuracy, robustness to noise and robustness to segmentation compared to existing methods.

## Graphical abstract

**Figure:**
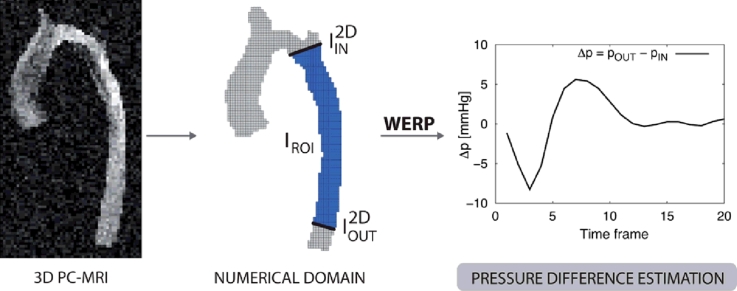

## 1 Introduction

Pressure differences, or pressure drops, measured over vascular segments are widely used clinically as biomarkers for a number of cardiovascular disorders (Baumgartner, Hung, Bermejo, Chambers, Evangelista, Griffin, Iung, Otto, Pellikka, Quiñones, 2009, Sawaya, Stewart, Babaliaros, 2012, Vahanian, Baumgartner, Bax, Butchart, Dion, Filippatos, Flachskampf, Hall, Iung, Kasprzak, Nataf, Tornos, Torracca, Wenink, 2007). A well-known example is aortic coarctation (CoA), where the pressure drop is used as a diagnostic metric to risk stratify patients undergoing surgery (Jenkins, Ward, 1999, Oshinski, Parks, Markou, Bergman, Larson, Ku, Mukundan, Pettigrew, 1997) and to evaluate patients after stenting (Tan et al., 2005). Other examples of pressure based metrics in the clinic include the transvalvular drop – an accepted metric to classify the severity of aortic valve stenosis (Baumgartner, Hung, Bermejo, Chambers, Evangelista, Griffin, Iung, Otto, Pellikka, Quiñones, 2009, De Bruyne, Manoharan, Pijls, Verhamme, Madaric, Bartunek, Vanderheyden, Heyndrickx, 2006, Feldman, 2006), the Left-Ventricle Outflow Tract (LVOT) pressure drop – used to define the guidelines for the treatment of Hypertrophic Cardiomyopathy (HCM) (Gersh et al., 2011), and the transstenotic pressure difference in the coronary artery – used to quantify the Fractional Flow Reserve (FFR) (Deng et al., 2014).

The measurement of pressure differences in current clinical guidelines is based on catheter measurements (Feldman, 2006, Konecny, Khanna, Novak, Jama, Zawadowski, Orban, Pressman, Bukartyk, Kara, Cetta, Borlaug, Somers, Reeder, 2014) or echocardiographic Doppler recordings (Bach, 2010, Firstenberg, Greenberg, Smedira, Prior, Scalia, Thomas, Garcia, Michael, Smedira, Thomas, 2000, Fyfe, Currie, Seward, Tajik, Reeder, Mair, Hagler, 1984, Labovitz, Ferrara, Kern, Bryg, Mrosek, Williams, 1986, Zhang, Nitter-Hauge, 1985). Pressure catheterization has seen significant improvement in terms of probe sensitivity (de Vecchi, Clough, Gaddum, Rutten, Lamata, Schaeffter, Nordsletten, Smith, 2014, Garcia, Carrozza, 2007, Iwasaki, Kusachi, 2009) and surgical administration, making it the gold standard in pressure drop measurement. However, despite its advantages, application of pressure catheterization is limited to specific cohorts of patients due to its intrinsic invasiveness and associated risks. To broaden the base of patients who could benefit from these assessments, non-invasive evaluation using Doppler echocardiography was developed. Applying this modality, the pressure difference is estimated from the peak velocity magnitude acquired along the direction of an ultrasound beam through a simplified Bernoulli formulation (Hatle, Brubakk, Tromsdal, Angelsen, 1978, Oshinski, Parks, Markou, Bergman, Larson, Ku, Mukundan, Pettigrew, 1997). While useful for patient stratification, the accuracy of this approach is limited by operator dependence and the mathematical assumptions which rely on neglecting transient effects and viscous losses on the flow (Holen, Simonsen, 1979, Laske, Jenni, Maloigne, Vassalli, Bertel, Turina, 1996, Zhang, Nitter-Hauge, 1985).

Working with the same Doppler Echocardiography data, pressure differences estimation has been improved by the use of Euler equations, as used in the characterisation of diastolic performance (Bermejo, Antoranz, Yotti, Moreno, Garcia-Fernandez, 2001, Greenberg, Vandervoort, Firstenberg, Garcia, Thomas, Neil, Firstenberg, 2001, Yotti, Bermejo, Antoranz, Rojo-Álvarez, Allue, Silva, Desco, Moreno, García-Fernández, 2004). This approach benefits from high temporal resolution of the data, but neglects the effects related to advective acceleration out of the line of insonation as well as to viscous dissipation. Doppler acquisitions are also dependent on the ability of the operator to detect the blood flow direction. All these factors have motivated continued research to improve robustness, accuracy and operator independence.

Recent advances in Magnetic Resonance Imaging (MRI) and Echocardiography have allowed the acquisition of velocity data in three-dimensional space and time (Deng, Fan, Xie, He, Natsuaki, Jin, Bi, An, Liu, Zhang, Fan, Li, 2014, Herment, Besson, Frouin, 2008, Markl, Wallis, Brendecke, Simon, Frydrychowicz, Harloff, 2010, Nielsen, Powell, Gauvreau, Marcus, Prakash, Geva, 2005). Ongoing research efforts have produced a number of different techniques to estimate pressure differences using these images. Particularly, Four Dimensional Phase-Contrast MRI (4D PC-MRI) data enables the solution of the Poisson Pressure Equation (PPE), where pressure is derived explicitly as a function of the acquired velocity field (Bock, Frydrychowicz, Lorenz, Hirtler, Barker, Johnson, Arnold, Burkhardt, Hennig, Markl, 2011, Krittian, Lamata, Michler, Nordsletten, Bock, Bradley, Pitcher, Kilner, Markl, Smith, 2012), allowing the estimation of the convective effects in all spatial directions and the contribution of viscous dissipation (Lamata et al., 2014). This approach has been successfully applied for the estimation of the pressure in aortic coarctation (Riesenkampff et al., 2014). Building on these data-driven methods, reconstruction of the velocity field at the vascular walls (Donati et al., 2014) has been proposed to recover the viscous effects, and data-assimilation techniques attempted to overcome the limitations of data acquisition with physically-based simulations (de Hoon et al., 2014).

An alternative approach to estimate pressure differences in the vascular anatomy is based on 3D Computational Fluid Dynamics (CFD) simulations (Kim, Vignon-Clementel, Coogan, Figueroa, Jansen, Taylor, 2010, LaDisa, Figueroa, Vignon-Clementel, Jin Kim, Xiao, Ellwein, Chan, Feinstein, Taylor, 2011, Sankaran, 2012, Vignon-Clementel, Figueroa, Jansen, Taylor, 2010). In this case, patient specific geometric models are reconstructed from images such as computed tomography angiography and velocity boundary conditions are defined from flow measurements. Consequently, pressure and velocity are simulated over the cardiovascular model (Coogan, Humphrey, Figueroa, 2013, Xiao, Alastruey, Figueroa, 2014), providing detailed metrics of flow, pressure differences, wall shear stress, amongst others. While providing these detailed metrics, forward cardiovascular modeling based on CFD requires robust multi-scale approaches for boundary conditions (Formaggia, Gerbeau, Nobile, Quarteroni, 2002, Gresho, Sani, 1987, Vignon-Clementel, Figueroa, Jansen, Taylor, 2006), accurate anatomical definition and the solution of expensive, parallel simulations in a computer cluster.

In this work, we present a novel non-invasive semi-automatic method for the estimation of pressure differences based on the work-energy theorem. The formulation introduced benefits from simplicity and computational efficiency, requiring integrations and computations that can be executed directly from the image acquired using 4D PC-MRI or Echocardiography. Introducing the mathematics behind the method, we detail its application for cardiovascular flow data. We test the method on a series of in silico test cases with progressively increasing complexity, evaluating robustness to segmentation variability and noise. Subsequently, the proposed method is thoroughly compared with other available methods on an in silico CFD solution. Finally, the satisfactory performance of the method is demonstrated on 4D PC-MRI acquisitions on a cohort of 9 healthy patients, by comparing estimated aortic pressure differences to previously reported results obtained with a PPE-based approach (Lamata et al., 2014). We conclude by highlighting the benefits of the new approach and proposing possible improvements for translation of this technique into the clinic.

## 2 Methods

Starting from the work-energy principle, we derive the formula for the pressure difference over a vascular segment (Section 2.1). Subsequently, we detail its discrete formulation (Section 2.2) and pre-processing steps (Section 2.3) required to work with 4D PC-MRI data. Finally, we briefly review the formulation of the alternative methods that can be found in the literature (Section 2.4).

### 2.1 Pressure difference from fluid work energy

Pressure differences in a fluid system are related to the kinematics of the flow field. This relationship is described by the well-known Navier–Stokes equations where, in the absence of gravity, variations in pressure are balanced by fluid accelerations and viscous stresses. Using the conservation of mass and momentum for closed systems, the work-energy for an incompressible isothermal Newtonian fluid over a Region Of Interest (ROI) (*Ω*) with boundary *Γ* yields,

$$\begin{array}{ccc} & & \underset{\frac{\partial }{\partial t}K_{e}}{\underset{︸}{\frac{\rho }{2}\frac{\partial }{\partial t}\int_{\Omega }\left(v\cdot v\right)\;dx}}+\underset{A_{e}}{\underset{︸}{\frac{\rho }{2}\int_{\Gamma }{\left|v\right|}^{2}\left(v\cdot n\right)\;dx}}+\underset{H(p)}{\underset{︸}{\int_{\Gamma }p\;v\cdot n\;dx}}\\ & & \;-\underset{S_{e}}{\underset{︸}{\int_{\Gamma }\mu \left[D\left(v\right)\cdot n\right]\cdot v\;dx}}+\underset{V_{e}}{\underset{︸}{\frac{\mu }{2}\int_{\Omega }D\left(v\right):D\left(v\right)\;dx}}=0, \end{array}$$

where ***v*** represents the velocity, *p* the pressure, n is the normal vector on *Γ*, $D\left(\cdot \right)=[\nabla \left(\cdot \right)+\nabla {\left(\cdot \right)}^{T}],$ and *ρ* and *μ* as the fluid density and dynamic viscosity. Here, $\frac{\partial }{\partial t}K_{e}$ is the temporal derivative of the kinetic energy within *Ω, A_e_* the advected energy rate describing the energy transfer due to the physical movement of a fluid in and out of *Ω* and *V_e_* is the rate of viscous dissipation. *H*(*p*) and *S_e_* represent energy inputs to the fluid system, the hydraulic power and the shear energy rate, respectively. Here we assume that the boundary of the *Ω* can be written as $\Gamma =\Gamma_{i}\cup \Gamma_{o}\cup \Gamma_{w},$ where *i, o* and *w* indicate contributions from the vessel inlet, outlet and walls surface. We refer to Taber (2004) and Appendix A for the mathematical details behind the work-energy principle derivation.

Starting from this work-energy balance, as a first approximation, we ignore the contribution to the advected energy *A_e_* from the lateral walls *Γ_w_*, as velocities are small in the near-wall regions compared to the core blood flow (Taylor, Figueroa, 2009, Xiao, Humphrey, Figueroa, 2013, Xiao, Alastruey, Figueroa, 2014). Consequently, computations are limited to the inlet and outlet cross-sections, e.g.

(1) $$A_{e}=\frac{\rho }{2}\int_{\Gamma_{i}\cup \Gamma_{o}}{\left|v\right|}^{2}\left(v\cdot n\right)\;dx$$

Furthermore, we assume the pressure to be nearly constant on the inlet and outlet planes, making

(2) $$H\left(p\right)=p_{i}\int_{\Gamma_{i}}v\cdot n\;dx+p_{o}\int_{\Gamma_{o}}v\cdot n\;dx+\int_{\Gamma_{w}}p\;v\cdot n\;dx.$$

When little or no compliance is present, |v·n|<<1 on the wall, the global mass balance compatibility condition yields,

(3) $$\int_{\Gamma }v\cdot n\;dx=\int_{\Gamma_{o}}v\cdot n\;dx+\int_{\Gamma_{i}}v\cdot n\;dx=0,$$

letting,

(4) H(p)=ΔpΛ,

where$\Delta p=p_{o}-p_{i}$ is the pressure difference between the outlet and inlet and $\Lambda =\int_{\Gamma_{o}}v\cdot n\;dx$ is a term accounting for the flux through surfaces, a term that can be expressed as a function of the inlet surface only by means of Eq. 3.

Regarding the shear energy *S_e_*, we consider the contribution over each boundary segment – inlet, outlet and wall – to be effectively zero. On inlet / outlet planes, this term contributes if there are significant gradients in the direction of the boundary normal. While these gradients can occur – particularly in bending or tapering vessels – they are extremely mild and effectively scaled away by the low viscosity of blood. This argument on the flow gradients cannot be assumed near the vessel walls, where a significant wall shear stress is induced. However, as this shear stress is principally orthogonal to the wall velocity (which predominantly dilates in the boundary normal direction), the contribution of these shear stresses to *S_e_* is assumed negligible.

With the assumptions above, the Work-Energy Relative Pressure (WERP) formulation to estimate the pressure difference based on energy contributions yields,

(5) $$\Delta p=-\frac{1}{\Lambda }\left(\frac{\partial }{\partial t}K_{e}+A_{e}+V_{e}\right).$$

From this equation, we observe that all RHS terms are directly derived from flow data, enabling the computation of the pressure difference. However, we also observe that this computation requires that |*Λ*| > 0 (e.g. that flow is observed through the vascular segment).

### 2.2 Computation from 4D PC-MRI

Let ***V***^*t*^ represent the velocity image acquired at time *t*, ***V***^*t*^(*i, j, k*) the velocity field evaluated at time *t* at the voxel (*i, j, k*) and *Δt* the discrete time step between two consecutive acquisitions. We discretize derivatives in Eq. 5 using a central finite difference method and estimate the pressure difference between inlet/outlet planes at time $t+\frac{1}{2}$ as

(6) $$\Delta p^{t+\frac{1}{2}}\;=\;-\frac{1}{\Lambda (V^{t+\frac{1}{2}})}\left(\frac{K_{e}\left(V^{t+1}\right)\;-\;K_{e}\left(V^{t}\right)}{\Delta t}\;+\;A_{e}\left(V^{t+\frac{1}{2}}\right)\;+\;V_{e}\left(V^{t+\frac{1}{2}}\right)\right),$$

where velocities at $t+\frac{1}{2}$ are approximated to second order accuracy $O(\Delta t^{2})$ by

(7) $$V^{t+\frac{1}{2}}=\frac{1}{2}\left(V^{t}+V^{t+1}\right).$$

**Fig. 1:**
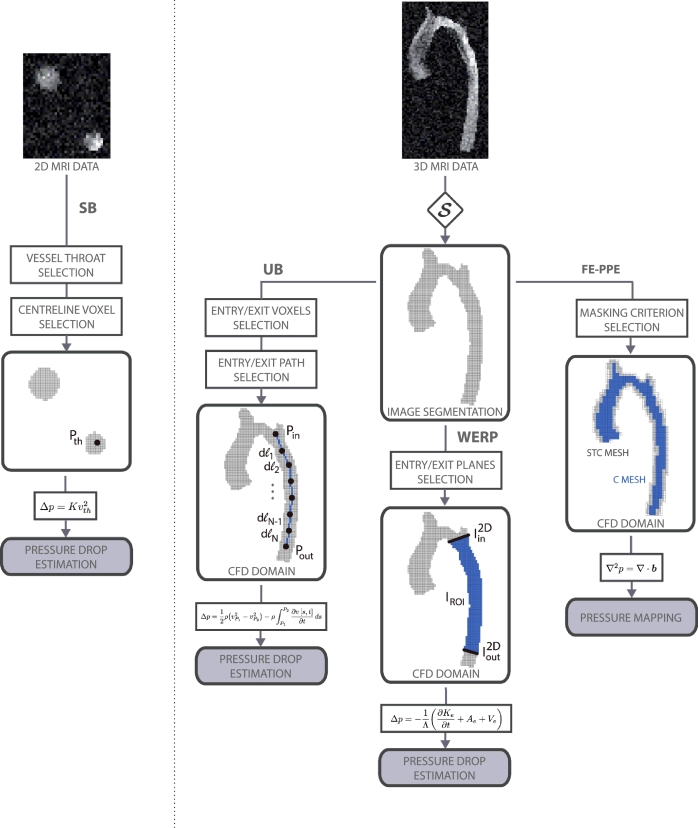
Schematic representation of the available methods to estimate time-dependent relative pressure non-invasively from PC-MRI, from left to right: Simplified Bernoulli (SB), Unsteady Bernoulli (UB), Work Energy-derived Relative Pressure (WERP), and Finite Element-based Poisson Pressure Equation (FE-PPE) on Core (C) mesh (in blue), and Static Tissue + Core (STC) mesh (in grey).

Computation of the WERP formulation terms is performed by integrating over a voxelized version of *Ω, I_ROI_*. Surface integrals are evaluated on the planes obtained by clipping the 3D mask to define inlet $I_{in}^{2D}$ and outlet $I_{out}^{2D}$ cross-sections (see Fig. 1) and the normal vectors ***N***_2*D*_(*i, j*). The discrete terms are then estimated from the image-based velocity field as,

(8) $$\begin{array}{ccc} \Lambda (V) & = & dS\sum_{\left(i,j\right)\in I_{out}^{2D}}M_{2D}\left(V\right)\left(i,j\right)\cdot N_{2D}\left(i,j\right),\\ K_{e}\left(V\right) & = & \rho \;dV\sum_{\left(i,j,k\right)\in I_{ROI}}{\left|M\left(V\right)\left(i,j,k\right)\right|}^{2},\\ A_{e}\left(V\right)\; & = & \;\frac{\rho }{2}dS\;\;\sum_{\left(i,j\right)\in I_{in}^{2D}\cup I_{out}^{2D}}\;\;{\left|M_{2D}\left(V\right)\left(i,j\right)\right|}^{2}\;\cdot \;\left(M_{2D}\left(V\right)\left(i,j\right)\cdot N_{2D}\left(i,j\right)\right),\\ V_{e}\left(V\right) & = & \frac{\mu \;dV}{2}\sum_{\left(i,j,k\right)\in I_{ROI}}D\left(V\right)\left(i,j,k\right):D\left(V\right)\left(i,j,k\right). \end{array}$$

where $dS=\Delta x^{2}$ and $dV=\Delta x^{3}$ are the pixel surface and voxel volume, respectively, based on the voxel length *Δx*. The discrete evaluation of all the contributions relies on the definition of the approximated velocity fields M(V) and $M_{2D}\left(V\right),$ obtained through averaging over the 3D mask and on the 2D planes defined above,

(9) $$\begin{array}{ccc} M(V)(i,j,k) & = & \frac{1}{2(1+q)}\sum_{n=o}^{q}(V\left(i+\delta_{n1},j+\delta_{n2},k+\delta_{n3}\right)\\ & & +\;V(i-\delta_{n1},j-\delta_{n2},k-\delta_{n3})),\\ M_{2D}\left(V\right)\left(i,j\right) & = & \frac{1}{2\cdot \max(1,q)}\sum_{n=o}^{\max(0,q-1)}(V\left(i+\delta_{n1},j+\delta_{n2}\right)\\ & & +\;V(i-\delta_{n1},j-\delta_{n2})). \end{array}$$

**Fig. 2:**
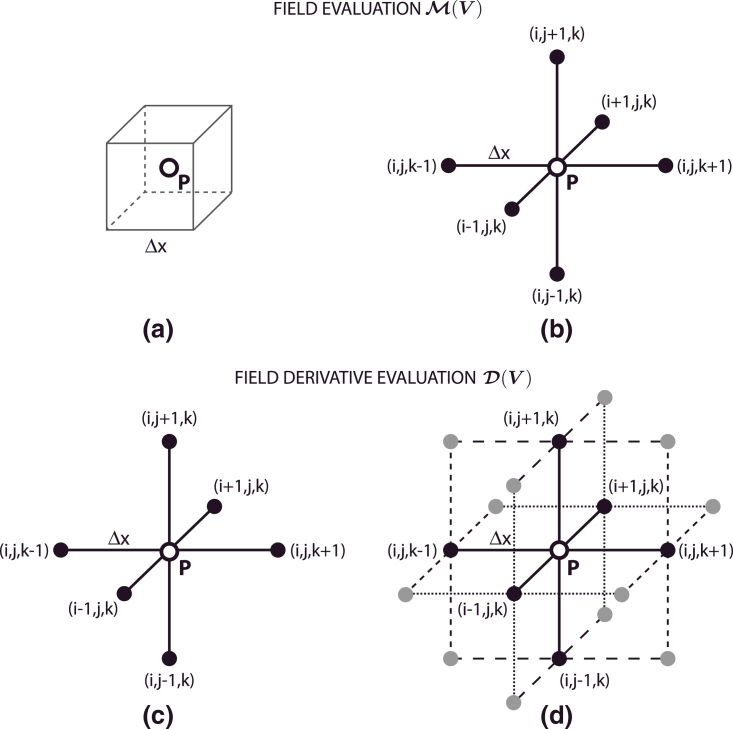
Schematic representation of the finite differences central stencils. Velocity field ***V*** evaluation at P=(i,j,k) through operator M(V) using standard (a) and filtered approach (b) and velocity field ***V*** derivative evaluation at P=(i,j,k) through operator D(V) using standard (c) and filtered central differences approach (d).

In the above, *δ_ij_* is the Kronecker delta and *q* is a parameter used to smooth the underlying data based on $O(\Delta x^{2})$ approximations to the velocity value (see Fig. 2). If q=0,M(V)(i,j,k)=V(i,j,k) and $M_{2D}\left(V\right)\left(i,j\right)=V\left(i,j\right)$ return the velocity measured at the voxel (*i, j, k*) and (*i, j*), respectively. Alternatively, if q=3, the measurement of the velocity field is taken as a weighted sum of $O(\Delta x^{2})$ approximations based on neighboring voxel measurements, effectively averaging out potential artefacts due to noise.

Similarly, in Eq. 8, the discrete tensor D(V) is calculated as,

(10) $$D\left(V\right)\left(i,j,k\right)=(G\left(V\right)\left(i,j,k\right)+G\left(V\right){\left(i,j,k\right)}^{T}),$$

where G(V) is a velocity gradient tensor defined as,

(11) $$G_{mn}\left(V\right)\left(i,j,k\right)=\frac{1}{2\cdot \max(1,q)}\sum_{r=0}^{q}\left(1-\delta_{rn}\right){\tilde{D}}_{n}^{r}V_{m}\left(i,j,k\right),$$

where

(12) $$\begin{array}{ccc} {\tilde{D}}_{n}^{r}V_{m}\left(i,j,k\right) & = & \frac{1}{2}(D_{n}V_{m}\left(i+\delta_{r1},j+\delta_{r2},k+\delta_{r3}\right)\\ & & +\;D_{n}V_{m}\left(i-\delta_{r1},j-\delta_{r2},k-\delta_{r3}\right)), \end{array}$$

and

(13) $$\begin{array}{ccc} & & D_{n}V_{m}\left(i,j,k\right)\\ & & \;=\frac{V_{m}\left(i+\delta_{n1},j+\delta_{n2},k+\delta_{n3}\right)-V_{m}\left(i-\delta_{n1},j-\delta_{n2},k-\delta_{n3}\right)}{2\Delta x}. \end{array}$$

Again, if q=0 velocity gradients are approximated by second order central differences centred at the voxel (*i, j, k*). Imposing q=3, a filtered approach is adopted, where the velocity derivative is approximated using weighted average of derivatives computed with second order central differences at neighboring voxels, therefore reducing noise contamination (see Fig. 2).

### 2.3 Required pre-processing

Prior to application in a clinical setting, a number of pre-processing steps are required. Field inhomogeneities and eddy currents (Chan, Von Deuster, Giese, Stoeck, Harmer, Aitken, Atkinson, Kozerke, 2014, Moussavi, Untenberger, Uecker, Frahm, 2014, Rohde, Barnett, Basser, Marenco, Pierpaoli, 2004) are corrected (1) using the pre-processing tools outlined in Bock et al. (2011). Subsequently, a binary mask *I_ROI_* is defined (2), based on a thresholding of the velocity magnitude calibrated by the maximum velocity *V_max_* (including voxels with a velocity magnitude greater than $S\;V_{max},$ with S being the segmentation thresholding parameter). Inlet and outlet points are manually selected by the user (3) depending on the clinical problem under investigation. A skeletonisation of the binary mask is then used to define the inlet and outlet planes perpendicular to the vessel (4). As a result of this process, the binary masks of the raw 3D image and the inlet/outlet planes needed for the WERP computation are defined. Within this work, the image acquisition process was mimicked in silico for the validations tests presented in Results 3.1, 3.2 and 3.3. Simulated PC MRI images were subsequently processed following (2)-(4) prior to application of the WERP method. On the contrary, the complete procedure described above (1)-(4) was followed to analyze the real cases presented in Results 3.4.

### 2.4 Pressure estimation from 4D PC-MRI: other approaches

To evaluate the performance of the WERP approach, we compared it to a selection of currently available non-invasive pressure differences estimation techniques. Specifically, in this paper we considered Simplified Bernoulli (SB), Unsteady Bernoulli (UB) and Finite Element-based Poisson Pressure Equation (FE-PPE) methods. We refer to Fig. 1 for a schematic representation of the workflow required for each of these techniques.

Starting with SB (Oshinski et al., 1997), the steady pressure difference in mmHg is computed as $\Delta p=Kv^{2},$ where *v*[m/s] is the throat velocity and *K*[mmHg s^2^/m^2^] is the loss or Bernoulli coefficient, which is usually taken as 4.0. Here, the main assumption is that viscous stresses are negligible compared to advective and kinetic contributions. This approach is used on PC-MRI or Doppler Echocardiography images by detecting the location where vessel narrowing is observed and selecting a pixel on the centreline of the throat cross-section (*i, j*) (pixel locations in the plane). Subsequently, the pressure difference at time $t+\frac{1}{2}$ can be defined from a velocity image as,

(14) $$\Delta p^{t+\frac{1}{2}}=-4{\left(V^{t+\frac{1}{2}}\left(i,j\right)\cdot N\right)}^{2},$$

where $V^{t+\frac{1}{2}}\left(i,j\right)$ is the highest velocity, ***N*** is the inflow/outflow direction, and (*i, j*) the pixel with peak throat velocity.

The UB formulation (Firstenberg et al., 2000b) builds from SB by incorporating the additional contributions due to inertial acceleration, defining the pressure difference in mmHg as,

(15) $$\Delta p=\frac{1}{2}\rho \left(v_{P_{in}}^{2}-v_{P_{out}}^{2}\right)-\rho \int_{P_{in}}^{P_{out}}\frac{\partial v\;[s,t]}{\partial t}\;ds,$$

where *P_in_* and *P_out_* are the upstream and downstream points defined along the aorta (see Fig. 1). The flow path is defined by the curvilinear coordinate *s* and *v*[m/s] is the projected velocity in the path direction. Inlet and outlet points *P_in_* and *P_out_* were defined from the image obtained after segmentation based on the intensity thresholding parameter S. Subsequently, the path was defined down the axis of the vessel (see Fig. 1) by selecting a series of N+1−sampling voxels $\left\{P_{1},\ldots P_{N+1}\right\}$ (with $d\ell_{i}=∥P_{i}-P_{i+1}∥$ denoting the distance between voxels). Following this formulation, the pressure difference at time $t+\frac{1}{2}$ yields^2^,

(16) $$\begin{array}{ccc} \Delta p^{t+\frac{1}{2}} & = & \frac{1}{2}\rho (|V^{t+\frac{1}{2}}\left(P_{in}\right){|}^{2}-{\left|V^{t+\frac{1}{2}}\left(P_{out}\right)\right|}^{2}\\ & & -\sum_{i=1}^{N+1}\left(\frac{V^{t+1}\left(P_{i}\right)-V^{t}\left(P_{i}\right)}{\Delta t}\right)\left(d\ell_{i}+d\ell_{i-1}\right)). \end{array}$$

We also compared WERP results with the time-dependent pressure difference estimated using the FE-PPE approach. The governing equation

(17) $$\nabla^{2}p=\nabla \cdot \left(\frac{\partial v}{\partial t}+\left(v\cdot \nabla \right)v+\mu \Delta v\right)$$

is discretised using a Galerkin finite element formulation (Krittian et al., 2012) using measured velocities to compute the unknown relative pressure field *p*. In this work, a quadratic mesh built on regular hexahedral elements was generated directly from the image, setting each voxel as a Degree Of Freedom (DOF) of the discrete mesh. The definition of the computational domain is thus based on cube elements of size 3 × 3 × 3 voxels. This led us to consider two inclusion criterions for the FE-PPE mesh, since the original paper does not specify this implementation detail: a valid element has all its 27 voxels or at least one voxel belonging to the segmentation mask, respectively defining a Core (C) mesh that neglects the boundary of the vessel lumen, or a mesh that includes static tissue, called Static Tissue + Core (STC) mesh. From the pressure field computed on both the grids, the pressure difference between the clipping planes used in the WERP procedure was evaluated.

## 3 Results

In this section, the performance of the WERP method is evaluated and compared with SB, UB and FE-PPE formulations. A preliminary in silico test in a straight pipe with Poiseuille steady flow was used to verify the WERP approach and to illustrate the impact of applying a standard or a filtered central differences stencil (Section 3.1). Further verification tests are presented in Section 3.2, where a time-space convergence analysis is performed in a pulsatile flow field. In Section 3.3, all methods are compared against CFD results from a patient-specific model of a human aortic coarctation, where we also examine the sensitivity of the WERP method to the image segmentation process. Finally, we test the performance of the method on 4D PC-MRI acquisitions on 9 healthy subjects, by comparing estimated pressure differences with reported results obtained with a FE-PPE based formulation (Lamata et al., 2014).

### 3.1 Laminar steady flow and noise reduction

**Fig. 3:**
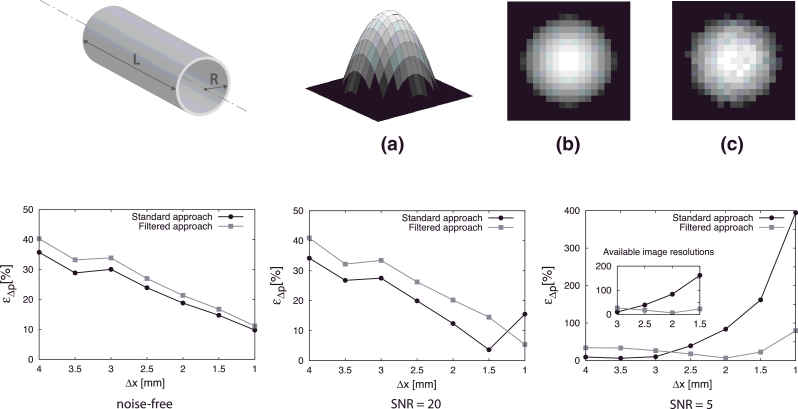
Validation of the WERP method on an in silico image data derived from Poiseuille flow. The dimensions of the cylindrical straight phantom (top left) are length L=10cm and radius R=1.5cm. Peak velocity $V_{max}=1\;m/s,$ blood density $\rho =1060\;\mathrm{kg}/m^{3}$ and dynamic viscosity μ=0.004Pa·s. Top images: visualization of the 3D analytical velocity profile (a), noise-free in-plane image (b) and noisy in-plane image (c). Bottom plots: pressure difference percentage relative error ε_*Δp*_ as a function of image resolution on a noise-free (left), low-noise SNR=20 (center) and high-noise SNR=5 (right) cases; average value over 100 simulation tests. Effect of standard (solid black line) and filtered stencil (solid grey line) with focus on currently available image resolutions.

The WERP method was first applied to an analytic laminar steady flow case. The purpose of this test was two-fold: first, to verify the accuracy of the method and second, to investigate the impact of enhanced filtered stencils presented in Fig. 2 on approximating the field and its spatial derivatives in the presence of noise. The pressure difference obtained using the WERP method was evaluated over an in silico phantom of a cylindrical straight pipe presented in Fig. 3. The vessel radius *R* and length *L*, density *ρ* and viscosity *μ* were chosen to be representative of those in the thoracic aorta.

The image acquisition process was simulated with increased image resolutions ranging from Δx=4mm to Δx=1mm isotropic. Pressure differences obtained by WERP using standard and filtered central approaches were compared against analytically derived pressure differences from Poiseuille theory. The comparison was quantified using the percentage relative error ε_*Δp*_,

(18) $${ɛ}_{\Delta p}=\frac{\left|\Delta p-\Delta p_{P}\right|}{\Delta p_{P}}\times 100,$$

where *Δp* is the pressure difference estimated with WERP method and $\Delta p_{P}=4\mu LV_{max}/R^{2}$ is the analytical solution.

To investigate the impact of noise on the pressure difference solution we performed three different tests, firstly comparing the standard and filtered approaches on a noise-free case, then introducing two levels of noise. Based on clinically reported Signal-to-Noise Ratios (SNR) for PC-MRI acquisitions in the human aorta – ranging from 10 to 25 (Friman et al., 2011) and from 10 to 50 depending on the use of contrast and on the magnet field strength (Hess et al., 2014) – we defined a low-noise level SNR = 20 and an high-noise level SNR = 5 to also test the method in the most restrictive situation. We assumed velocity noise to follow a random Gaussian distribution in each component (Gudbjartsson and Patz, 1995), with standard deviation *σ_N_* computed from the SNR (Drexl, Mirzaee, Harloff, Hullebrand, Hennemuth, Hahn, 2013, Lee, Pike, Pelc, 1995) as,

(19) $$\sigma_{N}=\frac{\sqrt{2}}{\pi }\frac{V_{ENC}}{SNR},$$

with the velocity encoding $V_{ENC}=V_{max}$. We obtained standard distributions $\sigma_{N}=2.25\%\;V_{ENC}$ and $\sigma_{N}=9\%\;V_{ENC}$ in the high- and low- noise level cases, respectively.

Results in Fig. 3 illustrate that the application of the standard central differences stencil is preferable in the noise-free or low-noise level configurations, with the exception of the highest spatial resolution tested Δx=1mm, where averaging over an enlarged cluster of voxels is beneficial, leading to a 5% maximum error. In the high-noise level case, performance of the standard central differences stencil is good for the largest resolutions, with a 10% maximum error with Δx=3mm, but the filtered approach shows a clear improvement of the estimate of pressure differences for the commonly used Δx=2mm image resolution, also leading to improved results for higher resolutions, which are typical for Steady-State Free Precession (SSFP) MRI acquisitions.

### 3.2 Transient flow verification and convergence analysis

**Fig. 4:**
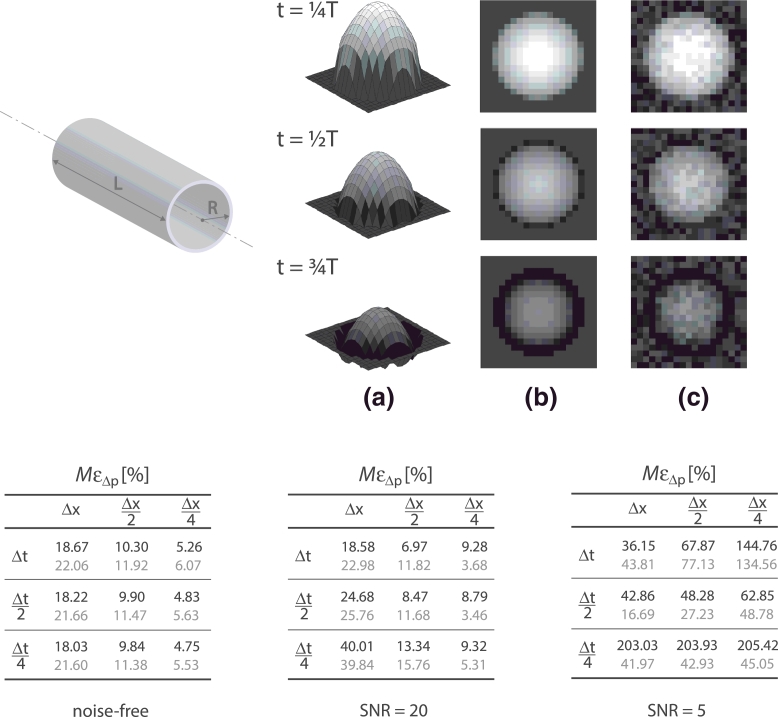
Convergence analysis of the WERP method on an in silico image data derived adding a Womersley and a Poiseuille flow solutions. The dimensions of the cylindrical straight phantom (top left) are length L=10cm and radius R=1.5cm. Peak velocity $V_{max}=1\;m/s,$ blood density $\rho =1060\;\mathrm{kg}/m^{3}$ and dynamic viscosity μ=0.004Pa·s. Top images: Visualization of the 3D analytical velocity profile at different phases (a), noise-free in-plane image (b) and noisy in-plane image (c). Velocity profiles are shown at different time frames during the simulated cardiac cycle with duration *T*. Bottom tables: Pressure difference maximum percentage relative error over time *M*ε_*Δp*_ as a function of space and time resolution, Δx=4mm and Δt=T/8s on a noise-free (left), low-noise SNR=20 (center) and high-noise SNR = 5 (right) cases; average value over 100 simulation tests. Effect of standard (black) and filtered stencil (grey).

To assess the spatiotemporal convergence of the WERP approximation in a more physiological setting, a pulsatile flow study was conducted on the in silico phantom presented in Section 3.1. The flow field was obtained as a linear combination of Poiseuille and Womersley – with a single pulsatile frequency – velocity profiles to better reproduce the unsteady features of the blood flow in the large vessels, as presented in Fig. 4. As in Section 3.1, a noise-free, a low-noise level SNR=20 and high-noise level SNR=5 conditions were replicated to also investigate the effects of noise and noise filtering. Again, each test was repeated 100 times to minimize spurious noise effects. We analyzed WERP results under improved spatiotemporal image resolution, varying the voxel dimension *Δx* ∈ [1, 4] mm isotropic and time step *Δt* ∈ [*T*/32, *T*/8] (where T=0.75s represents the pulsatile cycle duration). In the tables in Fig. 4, performance is evaluated in terms of the maximum percentage pressure difference error over time *M*ε_*Δp*_,

(20) $$M{ɛ}_{\Delta p}=\max_{t\in [0,T]}\;\frac{|\Delta p\left(t\right)-\Delta p_{PW}\left(t\right)|}{\Delta p_{PW}\left(t\right)}\times 100$$

where the analytic pressure difference $\Delta p_{PW}\left(t\right)=\Delta p_{P}+\Delta p_{W}\left(t\right)$ at time *t* is obtained by adding the steady Poiseuille reference solution *Δp_P_* and the time-dependent Womersley reference solution *Δp_W_*(*t*),

(21) $$\begin{array}{ccc} \Delta p_{P} & = & 4\mu LV_{max}/R^{2}, \end{array}$$

(22) $$\begin{array}{ccc} \Delta p_{W}\left(t\right) & = & \Delta p_{P}\cdot \exp\left(i\omega t\right), \end{array}$$

where ω=8.37rad/s is the angular frequency of the oscillations.

The results show expected convergence with spatiotemporal refinement in the noise-free configuration, with a minimum error around 5% for *Δx*/4 and *ΔT*/4. In the low-noise case, spatiotemporal convergence is achieved with a filtered stencil approach, which also shows beneficial effects in the high-noise case at all resolutions analyzed, with a 75% maximum error reduction compared to the standard approach at the highest spatiotemporal resolution.

### 3.3 Testing WERP on synthetic clinical data

**Fig. 5:**
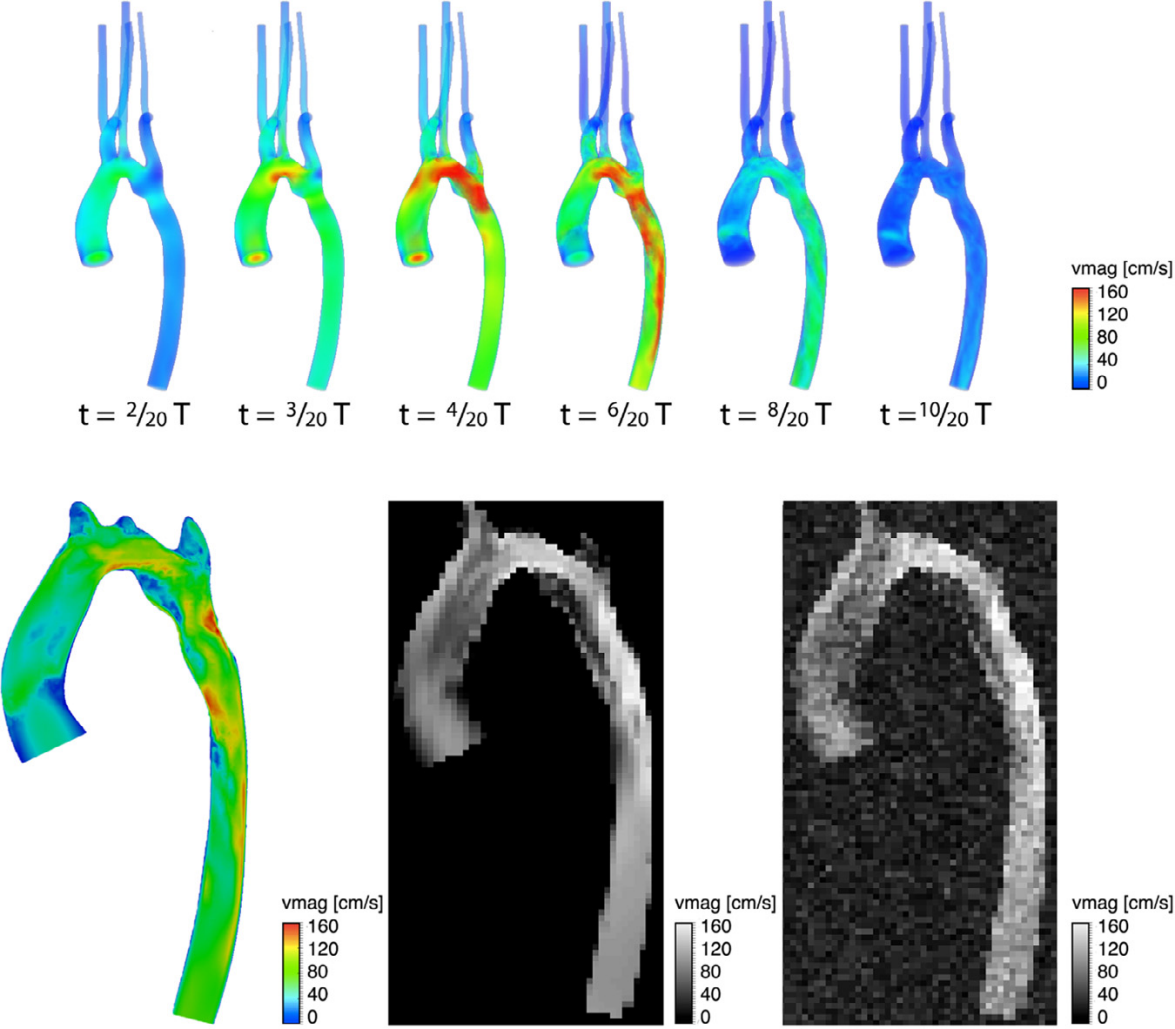
CFD simulation of a patient-specific model of a human aortic coarctation. Top images: volume-render view of the velocity magnitude field at different phases. Bottom images: axial plane visualizations of the intensity image at time t=6/20T (left), acquired noise-free image with Δx=2mm (centre) and acquired noisy image with Δx=2mm and simulated Gaussian noise with SNR=5 (right).

In order to verify the method on a more realistic case, we used the CFD simulation of haemodynamics in patient-specific model of a human aortic coarctation (see Fig. 5). The arterial compliance is accounted for using the Coupled-Momentum Method for Fluid-Solid Interaction deformable walls model (Figueroa et al., 2006). In this method, the fluid-solid interface is fixed, although its DOFs have non-zero velocities in general, as in transpiration-condition formulations (Deparis et al., 2003; Fernández and Tallec, 2003). This synthetic dataset provides simultaneous information on velocity and pressure over the entire CoA, and it is therefore a unique workbench for evaluating the performance of SB, UB, FE-PPE and WERP methods. Furthermore, the CFD pressure solution had been tested and verified against catheter pressure measurements (Sotelo et al., 2015).

In silico image data was synthesized from the simulations, sampling the cardiac cycle with duration T=0.75s (80 bpm) to provide 20 equally spaced time phases, with Δt=43ms. An image resolution Δx=2mm isotropic was used, and random Gaussian noise was added with SNR = 5, to simulate a worst case scenario. Blood density $\rho =1060\;\mathrm{kg}/m^{3}$ and dynamic viscosity μ=0.004Pa·s were also selected. An illustration of the workflow to mimic the acquisition process is presented in Fig. 5.

We extracted the computational domain from the noisy image generated prescribing a segmentation threshold $S=20\%\;{\bar{V}}_{max},$ where ${\bar{V}}_{max}=0.6\;m/s$ is the peak value of the velocity magnitude image obtained through averaging over the cardiac cycle. The pressure differences from the CFD simulation across arbitrarily defined locations of the descending aorta were compared to estimates obtained using the SB, UB, FE-PPE and WERP methods.

**Fig. 6:**
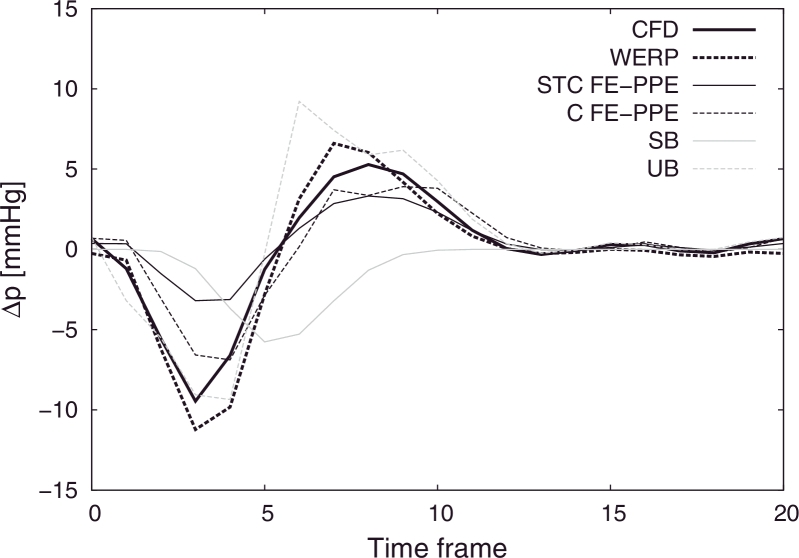
Comparison of pressure differences over the cardiac cycle computed with all available methods against benchmark solution from CFD simulations (solid black line): WERP (dashed black line), STC FE-PPE (solid dark grey line), C FE-PPE (dashed dark grey line), SB (solid light grey line) and UB (dashed light grey line).

**Fig. 7:**
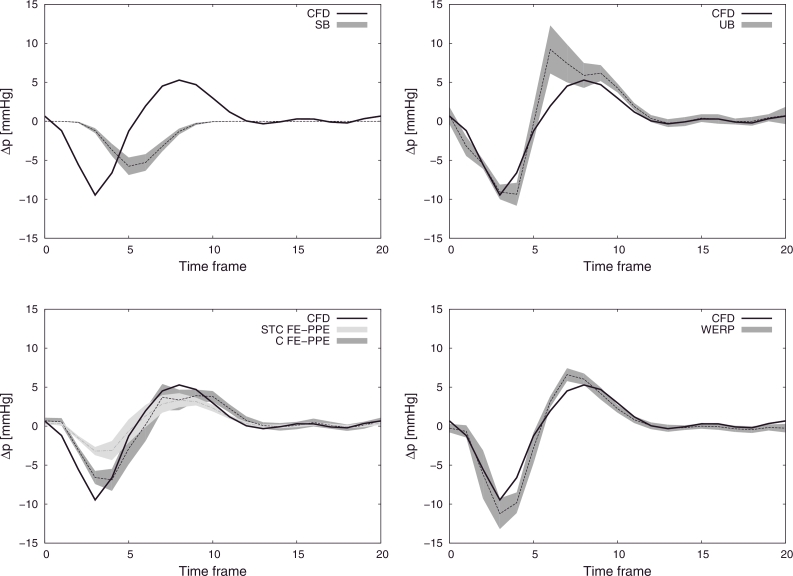
Sensitivity analysis to noise for the total pressure difference. CFD pressure difference over the cardiac cycle (solid black line) compared against SB (top left), UB (top right), STC FE-PPE (bottom left, light grey), C FE-PPE (bottom left, dark grey) and WERP (bottom right). Average pressure differences computed over 100 simulation tests (dashed lines) and 99% confidence intervals (grey filled curves).

In Fig. 6 the mean values of the pressure differences computed over 100 test repetitions with added noise are plotted over time, showing good accuracy with the WERP method, with 10% maximum overestimation of the pressure difference negative and positive peaks at the early systolic and diastolic phases, respectively. For completeness, Fig. 7 summarizes the sensitivity to noise of all the methods, presenting 99% confidence intervals over all tests.

**Fig. 8:**
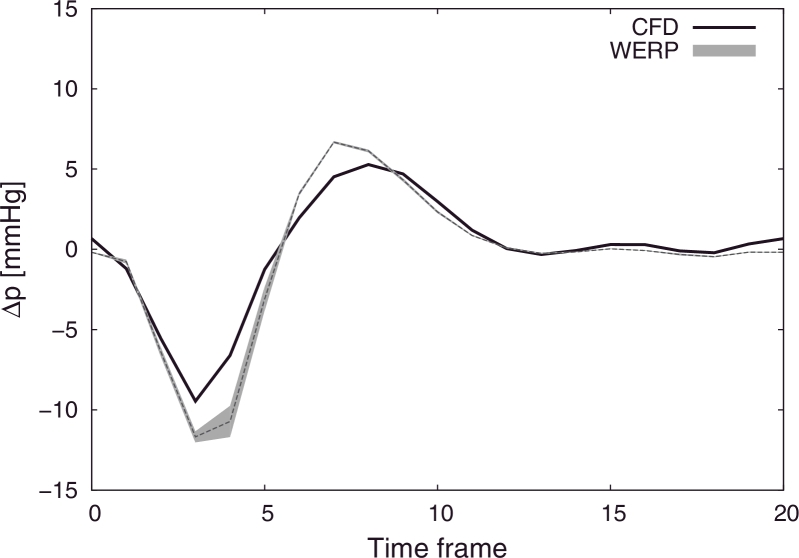
Sensitivity analysis to image segmentation process. Comparison of pressure differences over the cardiac cycle computed with WERP against benchmark solution from CFD simulations (solid black line). 99% confidence intervals of pressure differences computed over ROIs extracted using $S=20\%÷40\%\;{\bar{V}}_{max}$.

To further explore the robustness of the WERP method, we tested its sensitivity to the image segmentation process working on images synthesized from the CFD simulation. To this end, we computed the pressure difference over the ROIs generated by varying the segmentation threshold in the range $S=[20\%\;{\bar{V}}_{max},40\%\;{\bar{V}}_{max}]$ on an MRI image generated by selecting a given set of inlet/outlet planes (see Fig. 8).

### 3.4 Application of WERP on real clinical data

**Fig. 9:**
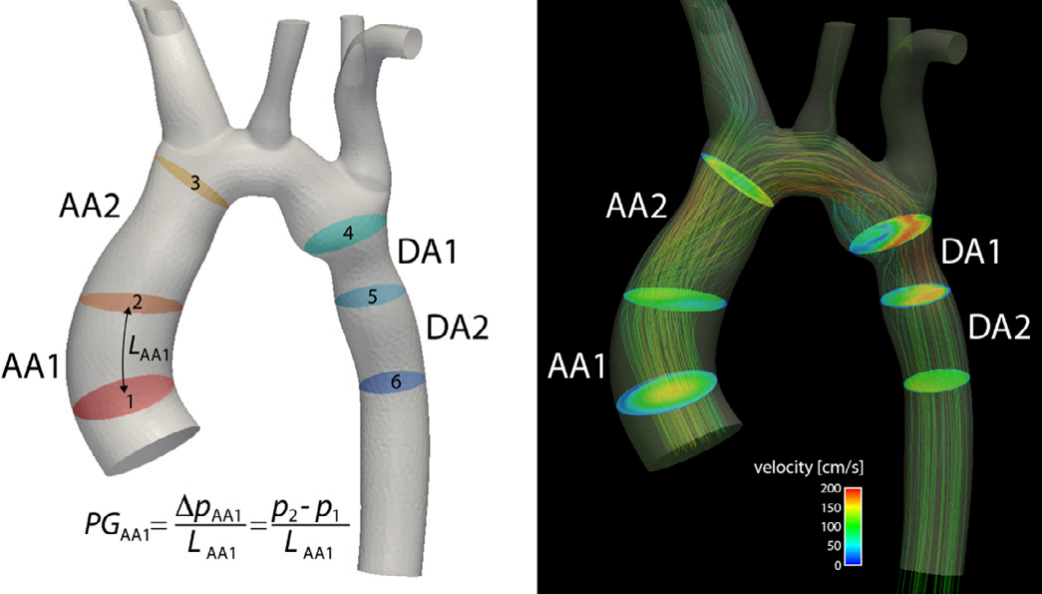
Definition of the four anatomical regions: ascending aorta - AA1 and AA2 - and descending aorta - DA1 and DA2. Left: illustration of the planes selected to define the anatomical regions. Pressure gradient with WERP method is computed as the pressure difference over a generic region defined by inlet and outlet planes divided by the aortic segment length *L*. (*L*_*AA*1_ in the example). Right: illustration of velocity magnitude surface plots and velocity streamlines during peak systole.

**Fig. 10:**
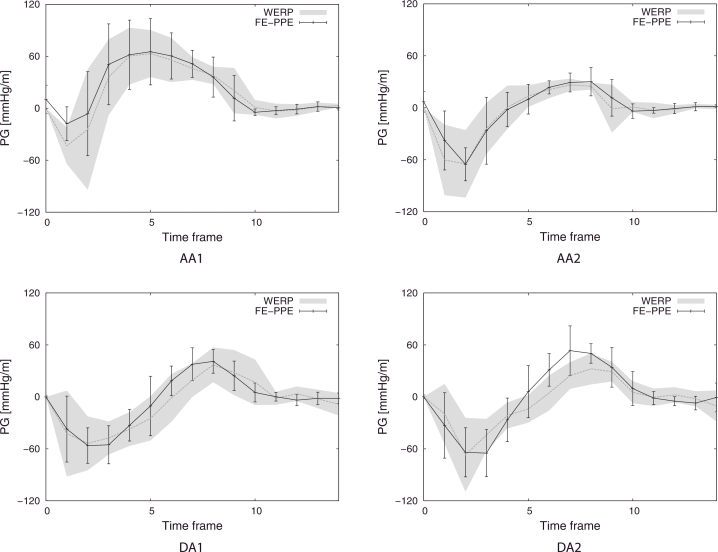
Average temporal profile of the pressure gradient in healthy subjects (*n*=9) in the four anatomical regions. Variability of the pressure gradients obtained with WERP (grey filled curve) and FE-PPE (errorbar plot) methods.

After in silico validation of the method, we applied it on real PC-MRI data of the thoracic aorta of a cohort of 9 healthy volunteers. Images were acquired using a 3T MR system (Trio, Siemens AG, Erlanden, Germany) with spatial resolutions of 1.25–1.77 × 1.25–1.77 × 3.2 mm^3^ (full details about the characteristics of these subjects and data acquisition parameters are provided in Lamata et al. (2014)). Pressure gradients were computed over four anatomical regions illustrated in Fig. 9: we divided the ascending aorta into *AA*1 - from the aortic valve (plane 1) to a plane defined by the pulmonary artery location (plane 2) - and *AA*2 - from plane 2 to the brachiocephalic artery (plane 3); similarly, we divided the descending aorta into *DA*1 - from the left subclavian artery (plane 4) to a plane defined by the pulmonary artery (plane 5) - and *DA*2 - from plane 5 to a plane defined at the same height of the aortic valve plane (plane 6). Then, we compared the WERP method performance against previously reported results (Lamata et al., 2014) obtained using the FE-PPE approach presented in (Krittian et al., 2012). Pressure gradients over the generic anatomical region *AR* were computed with WERP method as,

(23) $$PG_{AR}=\frac{\Delta p}{L_{AR}}=\frac{p_{AR,o}-p_{AR,i}}{L_{AR}},$$

where *L_AR_* is the anatomical region length estimated from the image and *p*_*AR,o*_ and *p*_*AR,i*_ the outlet and inlet pressures of the aortic segment, respectively. In Fig. 10, averaged temporal profiles of the pressure gradients and variability of the gradients over the 9 patients computed with WERP over the anatomical regions show good agreement with the results obtained using FE-PPE.

## 4 Discussion

In this study we introduced a novel method based on the work-energy principle for the computation of the pressure difference in cardiovascular compartments from dense velocity fields. The satisfactory accuracy and robustness exhibited by the method were thoroughly evaluated using in silico data.

### 4.1 Method convergence and accuracy

Spatial convergence was initially tested and verified in the steady flow case analyzed in Section 3.1, in noise-free or low-noise level conditions (see Fig. 3). On the contrary, in a high-noise level configuration, a key aspect on the WERP performance was the introduction of the filtering method presented in Section 2.2, whereby numerical stencils built over larger clusters of voxels are used to evaluate the field and its spatial derivatives, therefore preventing error amplification with higher image resolutions.

It must also be noted that the WERP formulation benefits from definition of the viscous dissipation term based on first order spatial derivatives, unlike the second order scheme utilized in the FE-PPE method (Krittian et al., 2012), thereby reducing high frequency noise amplification. In addition to this, the absence of gradients to estimate the advective contribution makes the proposed method an attractive choice in disease cases with jets and peak velocities larger than 2.5 m/s, which is the threshold that defines the appearance of a mild valve stenosis in clinical guidelines (Baumgartner et al., 2009).

Temporal and spatial convergence of the proposed method was tested in Section 3.2 using an analytical phantom with pulsatile flow, obtained as a combination of Womersley and Poiseuille solutions. Convergence was achieved in the noise-free case with both the approaches and in the low-noise level case by using a filtered approach only, which also partially limited the error amplification with spatiotemporal refinement in the high-noise level configuration.

Next, in Section 3.3 we further explored the power of the method, by testing its ability to capture the pressure difference along a human aortic coarctation dataset obtained from a patient-specific CFD simulation. The WERP averaged pressure differences compare well with the expected values from the simulations, demonstrating the consistency of our formulation (see Fig. 6).

In all these verification tests, to closely imitate the 4D PC-MRI clinical acquisition pipeline, we performed voxel rasterization with currently available resolutions (Markl et al., 2012) of the in silico geometry and flow field, which became the inputs to our algorithm. When noise was added, our method exhibited satisfactory robustness in comparison to other relative pressure estimation methods, as clearly visible in Fig. 7.

### 4.2 Comparative performance

Within the presented approach we introduced different features to overcome limitations observed in some of the existing pressure estimation methods. Indeed, to achieve clinical applicability, limited computational time is mandatory. The instantaneous pressure difference from the post-processed image data can be computed using the WERP method in approximately 1 min per frame with a standalone algorithm implemented in MATLAB R2013b^3^. This makes our method competitive against computational costs required with techniques based on unsteady Bernoulli formulation and more efficient than the FE-PPE technique, which had an approximate computational time of 10 min per frame using a Fortran 2008 implementation on the same machine. The WERP approach has also demonstrated a good agreement with FE-PPE working with real data (see Fig. 10).

In addition, the WERP method is based on a closed solution computed directly on the image velocity domain, with no need for iterative algorithms (Bock, Frydrychowicz, Lorenz, Hirtler, Barker, Johnson, Arnold, Burkhardt, Hennig, Markl, 2011, Ebbers, Farnebäck, 2009) or supplemental steps to define computational grids out of the image as in Kim et al. (2010); Krittian et al. (2012); Sankaran (2012). Here, the operator interaction is limited to the selection of planes only. The low sensitivity to the image segmentation process shown in Fig. 8 can be explained by the integral nature of our method: qualitatively, including or removing a single voxel in the computation is less crucial than doing so on a whole computational element. This makes the proposed approach intrinsically less sensitive to segmentation issues at the boundaries compared to FE-PPE based approaches (Donati et al., 2014). Furthermore, as shown in Fig. 6, the FE-PPE approach – despite being potentially able to provide accurate results at the expense of decreased robustness compared to the WERP method – is highly dependent on the mesh definition process, as demonstrated by the poor results obtained when part of the static tissue surrounding the vessel is included in the computational domain. Moreover, with the WERP method, the dependence on the integration path observed in unsteady Bernoulli approaches (Ebbers et al., 2001) is completely removed.

Comparative performance with CFD simulations for estimation of pressure differences was not attempted in this paper, but instead, results from these simulations were used for testing different methodologies. This workbench provides a ground truth both in terms of the flow velocity and pressure fields, allowing us to compare the performance of different pressure differences estimation algorithms. As all methods are based on simplified solutions to the Navier–Stokes equations, a good performance was expected – in the absence of added noise – on the synthetic datasets presented here. The comparison between image data-driven methods with model-based simulation approaches remains an area for further investigation.

### 4.3 Method limitations

The WERP method has been tested for a single vascular segment, with one inlet and one outlet plane. The analysis for a multi-branch model requires an adjustment of its mathematical formulation to account for branches along the ROI. While extension to multi-branch scenarios remains a future step, as most coarctations are located in the distal descending thoracic aorta, the current form could prove clinically useful.

Another limitation is that WERP has sub-optimal performance in flow regimes with net flow close to zero. In these circumstances – as the boundary flux *Λ* is the only term in the denominator of the WERP formulation (see Eq. 5) – small errors in its computation can introduce spurious amplification of computed pressure values. This effect did not occur in our in-silico workbench despite working with small diastolic flows and with realistic SNR values. But this might have an impact in real cases, for example when the ascending aorta experiences transitions from forward to retrograde flow. This could explain the discrepancy between the average temporal profiles of the pressure gradient computed over the 9 healthy subject in the anatomical region DA2 (see Fig. 10). In any case, this effect will not be present in the systolic events that are of current clinical diagnostic value.

**Fig. B.1:**
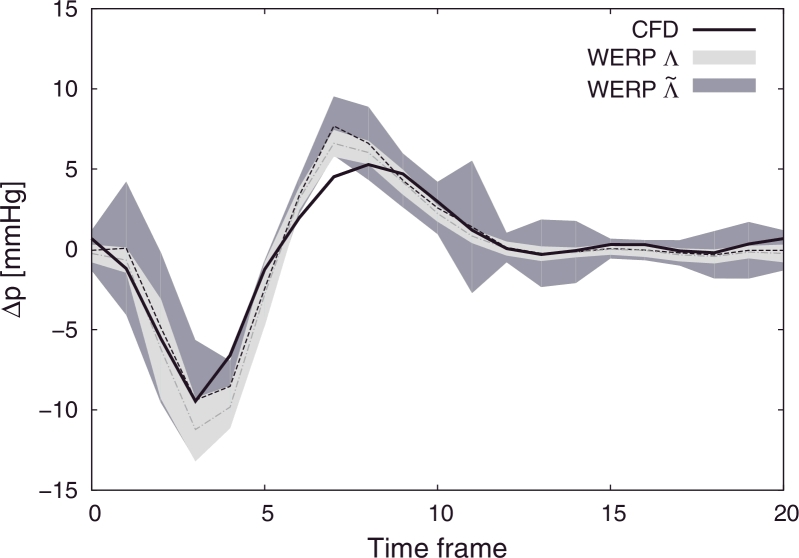
Comparison of pressure differences over the cardiac cycle computed using a boundary flux computed from the outlet plane of the ROI (*Λ*) or from the outlet and walls surface ($\tilde{\Lambda }$). CFD pressure difference (solid black line), WERP average pressure difference over 100 simulation tests (dashed black line) and WERP 99% confidence interval (grey filled color).

The negligible vessel wall compliance assumption of the WERP approach impacts the computation of *Λ*. On one hand, we have verified in the in silico aortic coarctation model that the inlet/outlet fluxes are at least two orders of magnitude larger than lateral flux through the arterial wall during systole, making the expected impact of this assumption minimal. In-vivo and in-vitro, this difference is likely to hold true during the systolic phase, but may no longer hold in the diastolic phase. On the other hand, to further explore this assumption, we included a wall compliance model to estimate the vessel boundary flux and tested the differences with the original method in Appendix B. However, as shown in Fig. B.1, this additional term does not improve results consistently. The reason is the locally low SNR close to the vessel wall and the presence of partial volume effects. It should be noted that, while including the wall compliance contribution would certainly improve the accuracy of results from a mathematical perspective, removing it completely does not compromise the final solution.

The computation of pressure differences requires an accurate estimation of temporal derivatives and spatial gradients of blood velocity. We have shown that in the presence of acquisition noise – uncorrelated between adjacent samples – increased temporal or spatial resolution amplifies the numerical derivatives error, leading to lack of convergence with spatiotemporal refinements. We introduced the filtered approach to allow averaging on multiple voxels and mitigate the error amplification, and showed its beneficial effect with available image resolutions and low SNR levels. Further work is nevertheless needed to identify the optimal filtering strategy for each image resolution, acquisition time and SNR.

To validate the performance of our method, we preliminarily used a CFD workbench instead of a real dataset. This choice is motivated by the need of having clean velocity data to which we could arbitrarily add noise, and of the complete understanding of the pressure difference, that would not be otherwise not be achievable experimentally. Moreover, the pressure solution from simulations – unlike real pressure measurements – can be further manipulated to obtain ground truth values for each of the pressure difference components (kinetic, advective and viscous), opening to potential applications to better characterize cardiovascular diseases (Lamata et al., 2014).

Finally, application of the method on real PC-MRI data was demonstrated on a cohort of 9 healthy volunteers, but ground truth data of the instantaneous pressure difference was not available. The reason is the difficult in-vivo acquisition of pressure data with sufficient accuracy, which can only be feasible with perfectly stable, located, calibrated and synchronized pressure wire sensors within the magnet of the MRI (Tyszka et al., 2000). Conventional fluid-filled catheters are not suitable due to the artefacts they introduce (de Vecchi et al., 2014). As a consequence of this experimental difficulty, previous studies do not compare the instantaneous pressure difference, but peak pressure values (Riesenkampff et al., 2014) or average pressure differences (Lum et al., 2007), or simplify the validation by the removal of the kinetic component in steady flow phantoms (Khodarahmi et al., 2010). Within this work, the proposed method was preliminarily tested against other methods on an ideal in silico workbench, but future work is required to confirm these results experimentally comparing to pressure sensor recordings.

### 4.4 Clinical perspectives

Recent research efforts provide compelling evidence that the analysis of blood flow dynamics can improve the management of cardiovascular diseases through flow-derived biomarkers. This is demonstrated by the analysis of the vortical flow in the ventricle (Pedrizzetti et al., 2014) or in the aorta (Bissell et al., 2013), the influence of wall shear stress on the endothelial function (Chiu and Chien, 2011), the estimation of flow energetics (Barker et al., 2014) and turbulence (Dyverfeldt et al., 2013), and the extraction of pressure gradients and its components (Lamata et al., 2014). A landmark recent study has provided initial evidence of the suitability of PC-MRI pressure estimation to assess the severity of aortic coarctation (Riesenkampff et al., 2014).

In this work we have conceptually built a bridge between the biomarkers of flow energetics and pressure differences, enabling a theoretical and practical assessment of their interaction and relative importance. The competitive accuracy and robustness compared to other methods makes the WERP approach an attractive alternative for the extraction of clinical biomarkers. The application of the proposed method to estimate pressure differences and its components from real MRI datasets from a cohort of healthy and diseased subjects with bicuspid aortic valves is currently undergoing.

## 5 Conclusions

In conclusion, this work demonstrates the potential applicability of the newly proposed approach to accurately estimate relative pressures from 4D PC-MRI data non-invasively, within clinically feasible times. Thorough validation and testing on progressively more complex cases showed increased robustness of the formulation compared to other pressure gradients estimation methods.

## Appendix A Mathematical derivation of the work-energy principle

The work-energy formulation used to estimate the total pressure difference across a region *Ω* extracted within the large human vasculature depends on the Navier–Stokes equations,

(A.1) $$\begin{array}{ccc} \rho \frac{\partial v}{\partial t}+\rho \nabla \cdot \left(vv\right)+\nabla p-\mu \nabla \cdot D\left(v\right) & = & 0, \end{array}$$

(A.2) $$\begin{array}{ccc} \nabla \cdot v & = & 0, \end{array}$$

which describe the momentum and mass conservations principles, respectively. Integrating Eq. A.2 over *Ω* and applying the divergence theorem results in the compatibility condition $\int_{\Gamma }v\cdot n=0$. To obtain the work-energy equation, we multiply Eq. A.1 by ***v*** and we integrate over the domain *Ω*, yielding

(A.3) $$\begin{array}{ccc} & & \frac{\rho }{2}\frac{\partial }{\partial t}\int_{\Omega }\left(v\cdot v\right)\;dx+\rho \int_{\Omega }\left[\nabla \cdot \left(vv\right)\right]\cdot v\;dx\\ & & +\int_{\Omega }\nabla p\cdot v\;dx-\mu \int_{\Omega }\left[\nabla \cdot D\left(v\right)\right]\cdot v\;dx=0. \end{array}$$

Applying integration by parts over the third and fourth term, and noting Eq. A.2, we observe that

(A.4) $$\begin{array}{ccc} & & \frac{\rho }{2}\frac{\partial }{\partial t}\int_{\Omega }\left(v\cdot v\right)\;dx+\rho \int_{\Omega }\nabla \cdot \left(vv\right)\cdot v\;dx+\int_{\Gamma }p\;v\cdot n\;dx\\ & & \;-\int_{\Gamma }\mu \left[D\left(v\right)\cdot n\right]\cdot v\;dx+\frac{\mu }{2}\int_{\Omega }D\left(v\right):D\left(v\right)\;dx=0. \end{array}$$

We may also apply integration by parts on the second term, reducing the volume integral to a surface integral. The work energy principle is then given for an incompressible isothermal Newtonian fluid over as,

(A.5) $$\frac{\partial K_{e}}{\partial t}+A_{e}+H\left(p\right)-S_{e}+V_{e}=0,$$

where

(A.6) $$\begin{array}{ccc} K_{e} & = & \frac{\rho }{2}\int_{\Omega }\left(v\cdot v\right)\;dx \end{array}$$

(A.7) $$\begin{array}{ccc} A_{e} & = & \frac{\rho }{2}\int_{\Gamma }{\left|v\right|}^{2}\left(v\cdot n\right)\;dx \end{array}$$

(A.8) $$\begin{array}{ccc} H(p) & = & \int_{\Gamma }p\;v\cdot n\;dx \end{array}$$

(A.9) $$\begin{array}{ccc} S_{e} & = & -\mu \int_{\Gamma }\left[D\left(v\right)\cdot n\right]\cdot v\;dx \end{array}$$

(A.10) $$\begin{array}{ccc} V_{e} & = & -\mu \int_{\Omega }D\left(v\right):D\left(v\right)\;dx \end{array}$$

## Appendix B Modeling vessel compliance

Within the WERP formulation, to deal with cases with compliance effects, we introduce an additional assumption that pressure is almost constant in every cross-sectional plane, which is reasonable as the absolute pressure in each cross section is typically much larger than the intra-cross-sectional variation. Introducing an axial coordinate *ξ* ∈ [0, 1], which is zero and one at the inlet an outlet, respectively, and assuming a near-linear variation along the vessel, the hydraulic power

(B.1) $$\begin{array}{ccc} H(p) & = & p_{i}\int_{\Gamma_{i}}v\cdot n\;dx+p_{i}\int_{\Gamma_{w}}\left(1-\xi \right)v\cdot n\;dx\\ & & +\;p_{o}\int_{\Gamma_{o}}v\cdot n\;dx+p_{o}\int_{\Gamma_{w}}\xi \;v\cdot n\;dx \end{array}$$

can be simplified as

(B.2) $$H\left(p\right)=\Delta p\;\tilde{\Lambda },$$

where $\tilde{\Lambda }$ is the boundary flux accounting for the vascular walls contribution

(B.3) $$\tilde{\Lambda }=\int_{\Omega_{i}}v\cdot n\;dx+\int_{\Omega_{w}}\xi \;v\cdot n\;dx.$$

Consequently, the pressure difference based on the work-energy principle in the presence of significant compliance effects can be estimated as

(B.4) $$\Delta p=-\frac{1}{\tilde{\Lambda }}\left(\frac{\partial }{\partial t}K_{e}+A_{e}+V_{e}\right)$$

The impact on the pressure difference computation is assessed by performing a comparison of the results obtained by computing the boundary flux based on the outlet plane only (*Λ*) or on the outlet and walls surfaces ($\tilde{\Lambda }$) of the vessel. In Fig. B.1, the total pressure difference over the cardiac cycle is captured with increased accuracy at the early systolic frames when adding information from the wall surface flux. However, the near-wall region is intrinsically affected by low SNR, which is therefore contaminating the computation of fluxes, especially in the diastolic frames. This is clearly shown by larger confidence intervals if the wall contribution is included in the boundary flux estimation.

## Footnotes

2 Note, we assume $d\ell_{0}=d\ell_{N+1}=0$.

3 The algorithm was implemented and tested on a Unix-based machine equipped with 3.4GHz Intel Core i7-2600 CPU.
